# Evaluating the effect of overharvesting on genetic diversity and genetic population structure of the coconut crab

**DOI:** 10.1038/s41598-020-66712-4

**Published:** 2020-06-22

**Authors:** Takefumi Yorisue, Akira Iguchi, Nina Yasuda, Yuki Yoshioka, Taku Sato, Yoshihisa Fujita

**Affiliations:** 1 Integrative Aquatic Biology, Onagawa Field Center, Graduate School of Agricultural Science, Tohoku University, 3-1 Mukai, Konori-hama, Onagawa, Oshika, Miyagi 986-2242 Japan; 2 Geological Survey of Japan, National Institute of Advanced Industrial Science and Technology (AIST), Tsukuba, Ibaraki, Japan, AIST Tsukuba Central 7, 1-1-1 Higashi, Tsukuba, Ibaraki 305-8567 Japan; 3 Department of Marine Biology and Environmental Science, Faculty of Agriculture, University of Miyazaki, Gakuenkibana-dai Nishi 1-1, Miyazaki, 889-2192 Japan; 4 Department of Bioresources Engineering, National Institute of Technology, Okinawa College, 905, Henoko, Nago, Okinawa 905-2192 Japan; 5 Research Center for Marine Invertebrates, National Research Institute of Fisheries and Environment of Inland Sea, Japan Fisheries Research and Education Agency, Momoshima, Onomichi, Hiroshima 722-0061 Japan; 6 Okinawa Prefectural University of Arts, 1-4, Shuri Tonokura-cho, Naha-shi, Okinawa 903-8602 Japan; 7 Present Address: Institute of Natural and Environmental Sciences, University of Hyogo, Yayoigaoka, Sanda, Hyogo 669-1546 Japan; 8 Present Address: Museum of Nature and Human Activities, Yayoigaoka, Sanda, Hyogo 669-1546 Japan

**Keywords:** Conservation biology, Molecular ecology

## Abstract

*Birgus latro* (coconut crab) is an edible crustacean that has experienced serious overharvesting throughout its whole habitat range; however, the negative effects of overharvesting on the genetic diversity within *B. latro* populations have not been elucidated. Here, we report sex ratio, body size, and genetic diversity in populations of *B. latro* in the Ryukyu Islands where large-male–biased overharvesting of *B. latro* has continued. In 2 of the study populations, the sex ratio was significantly skewed toward females, and in all of the study populations large males were rare, which we attributed to sex- and size-biased overharvesting. We found no differences in genetic diversity between small and large individuals, suggesting that genetic diversity, even among the large (i.e., old) individuals, may have had already been negatively affected by overharvesting. Continued monitoring of sex ratio, body size and genetic diversity are needed for effective management of the study populations.

## Introduction

Overharvesting drives loss of genetic diversity^1–3^. Once genetic diversity is lost from a population, it can be restored by genetic mutation or immigration of individuals from a population with high genetic diversity. However, the recovery of genetic diversity through mutation takes a long time, and although immigration from refugia is faster than mutation, the restoration of genetic diversity in isolated populations cannot be expected to occur through this means^3^. Therefore, conserving genetic diversity and increasing our understanding of gene flow patterns should be important goals for the effective management of fishery resources^3–5^.

Individual genetic diversity (individual heterozygosity) likely plays an important role in population sustainability because it is correlated with fitness. For example, a meta-analysis has shown that the correlation between the level of individual heterozygosity and fitness is small but significantly positive^6^, although outbreeding depression has been shown to cause the correlation to become negative^7^. In addition, many papers have reported significant positive relationships between individual heterozygosity of allozyme or neutral microsatellite markers and fitness-related traits in taxa such as fish^8–12^, mollusks^13,14^, crustaceans^15^, marine and terrestrial mammals^16–19^, and terrestrial plants^20^. It is therefore important to investigate genetic diversity to assess the sustainability of fishery resources.

High, long-term harvesting pressure often causes the average body size within a population to decrease^21^. Body size is a key factor that influences fitness in many taxa^22,23^. In males, larger individuals are stronger competitors for females^24^ and provide more sperm per ejaculation^25^, and in females, body size is positively correlated with number of eggs^26^, egg size^27^, and larval body size and starvation resistance^28^.

The coconut crab (*Birgus latro*) is a terrestrial hermit crab with a marine larval dispersal stage distributed in subtropical and tropical regions of the Indo-Pacific^29,30^. Recent report showed that this species predates other animals including birds and mammals, and can have strong impacts on prey behavior, abundance and community composition^31^. However, this species has suffered severe resource depletion throughout its entire distribution range due to overharvesting and habitat destruction^30–35^. Therefore, *B. latro* is currently categorized as “data deficient” in the International Union for Conservation of Nature’s Red List, and “vulnerable” in the Japanese Ministry of the Environment’s Red Data Book^36^. In Japan, *B. latro* is an important resource not only for food but also for culture and tourism^37^. However, selective harvesting of large males has resulted in the sex ratio of local populations becoming skewed toward females and the average male body size becoming miniaturized in places that have experienced high fishery pressure^38^. Recent studies have revealed that large-male–biased harvesting negatively affects populations in several ways^39^: it decreases male size-dependent reproductive potential [e.g., number of retained sperm^40^, number of possible mates^41^, and number of ejaculated sperm^42^], reduces the pool of suitable males due to females refusing to mate with males smaller than themselves^38^, which results in reductions of number of spawned eggs^43^ and larval qualities^44^]. Thus, it is likely that decreases of average body size within a population will have negative impacts on the population’s genetic diversity through massive decrease of effective population size.

**Figure 1:**
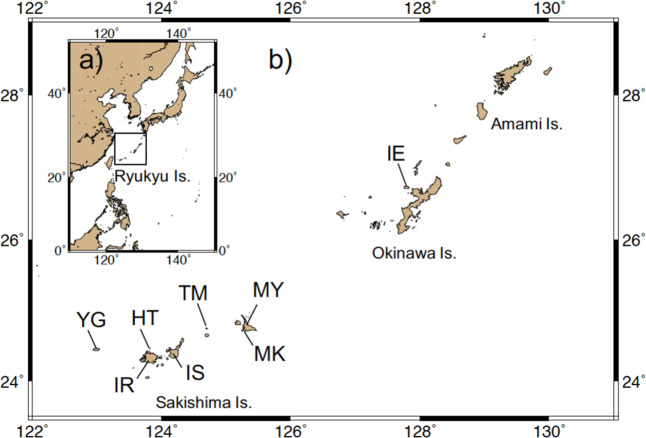
Map showing sampling localities of *B. latro* in the Ryukyu Islands. (**a**) A map of east Asia. (**b**) A map of sampling location of the present study in the Ryukyu Islands, Japan.

Recently, the use of multiplexed inter-simple sequence repeat (ISSR) genotyping by sequencing (MIG-seq)^45^ to analyze large numbers of genome-wide markers has become a useful tool for examining the genetic structure and diversity of populations at high temporal and spatial resolution^46^. Here, we examined sex ratio, thoracic length (as an index of body size), and genetic diversity (by COI gene and MIG-seq analyses) to elucidate the effects of overharvesting on population structure and genetic diversity as well as gene flow pattern among 8 populations (Fig. 1) of *B. latro* in the Ryukyu Islands, Japan.

## Results

### Sex ratio and body size

**Figure 2:**
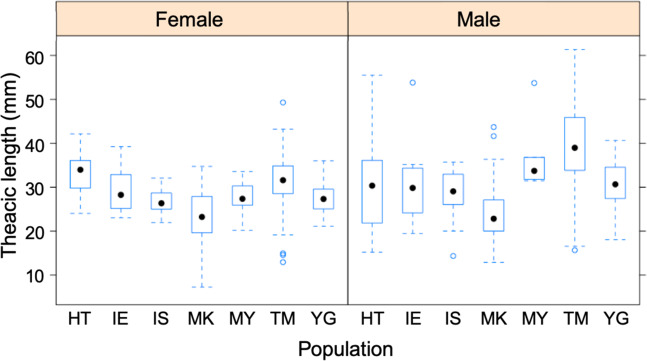
Box plot showing thoracic length of female (**a**) and male (**b**) *B. latro* individuals in each population.

In all study populations except Kurima (MK), the number of females was higher than that of males, although the difference was only significant in Ishigaki (IS) and Hatoma (HT) (Table 1). Female thoracic length in MK was significantly smaller than that in IE, TM, IS and HT. Male thoracic length in MK was significantly smaller than that in MY, TM, HT and YG (Fig. 2b; Table S1). In addition, male thoracic length in TM was significantly larger than that in IE, MK, IS, HT and YG (Fig. 2b; Table S1).

**Table 1:** Information of sampling locality, sampling month/year, sample size, sex ratio and 95% confidence interval of the sex ratio of *B. latro*.

Locality	Code	Island size (km^2^)	Population density (people / km^2^)	Sampling month/year	Sample size			Sex ratio	95% confidence interval
					COI	MIG-seq	Body size		
Ie	IE	22.8	3179	Oct 2015	23	11	36	0.56	0.38–0.72
Miyako	MY	158.9	4002	Aug 2015	13	5	13	0.62	0.32–0.86
Kurima	MK	2.8	1276	Aug 2015	24	14	114	0.44	0.35–0.53
Minna	TM	2.2	238	Jun 2014	15	7	235	0.56	0.48–0.63
Ishigaki	IS	222.3	2391	Jun 2014	17	7	50	0.66	0.51–0.79
Hatoma	HT	1.0	1809	Jun 2014	24	13	54	0.65	0.51–0.77
Iriomote	IR	289.6	106	Sep-Oct 2015	14	12	na	na	na
Yonaguni	YG	29.0	1150	Aug 2015	24	14	30	0.60	0.41–0.77

Population density denotes cumulated data of every 5 year from 1955 to 2015 ^64^. Sex ratio indicates proportion of number of female individuals in each population.

Correlation test with Spearman’s rank correlation coefficient showed no significant correlation between cumulative human population density and, body size and proportion of female individuals (Fig. S1).

### Genetic diversity and population genetic structure

#### COI gene analysis

**Figure 3:**
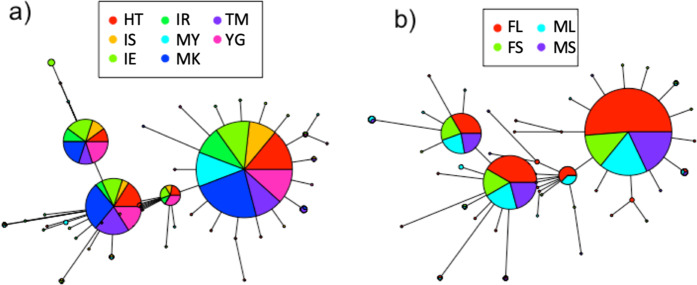
Haplotype network of *B. latro* showing frequency of mtDNA COI haplotypes in each population (**a**) and size class of both sexes (**b**). FL, large female; ML, large male, FS, small female; MS, small male.

We sequenced a 466-bp region of the mtDNA COI gene sequence in 154 individuals and detected 53 haplotypes. Four dominant haplotypes were found in most of the study populations (Fig. 3a). Population pairwise PhiPT was low overall (PhiPT < 0.018), and no significant differences were found among the populations (Table S2). MK and TM showed low genetic diversity compared with the other populations (Table S3). No marked difference of genetic diversity was detected between small and large individuals for either sex (Fig. 3b; Table S3).

Correlation test with Spearman’s rank correlation coefficient showed no significant correlation between cumulative human population density and, haplotype diversity and nucleotide diversity, (Fig. S1).

#### MIG-seq analysis

**Figure 4:**
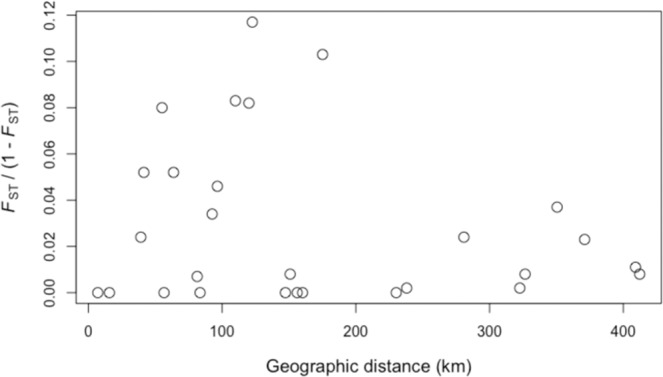
Relationship between pairwise population genetic distance inferred from MIG-seq SNP markers and geographical distance in *Birgus latro*.

**Figure 5:**
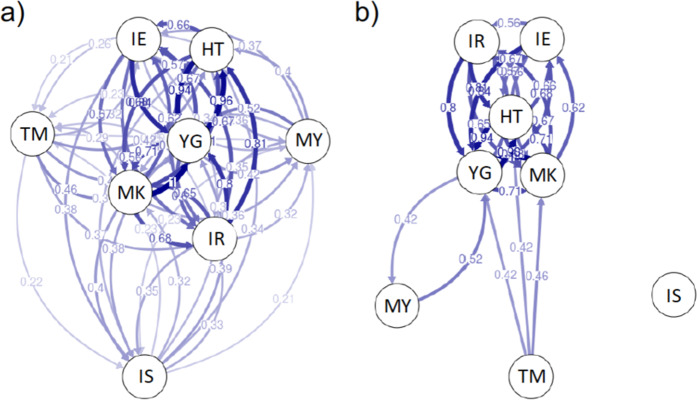
Directional relative migration networks of *B. latro* populations constructed with divMigrate using Nm. Values above 0.2 (**a**) and 0.4 (**b**) are shown.

**Figure 6:**
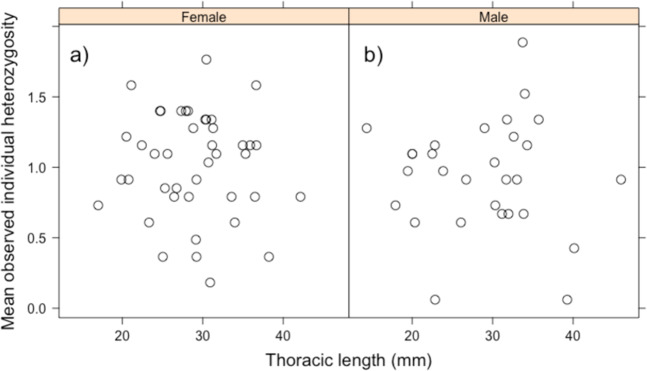
Plot showing body size and mean observed individual heterozygosity of female (**a**) and male (**b**) *B. latro*. Lines indicate linear regression.

We used 495 neutral genetic markers for the genetic analysis. TM and IS were significantly differentiated from both MK and YG (*F*_ST_ = 0.074–0.104, *P* < 0.01; Table S2). No significant relationship between genetic distance and geographic distance was detected by Mantel’s test (*R*^2^ = 0.042, *P* = 0.232; Fig. 4). Observed heterozygosity was low in TM (0.041) and I (0.042), and high in M (0.059), compared with that in the other populations (Table S3). Fixation index values were high in all populations (*F* = 0.294–0.532; Table S3). The directional relative migration network for the study populations indicated that IE, MK, HT, IR, and YG are core populations that have high gene flows among each other, whereas MY, TM, and IS are peripheral populations with low gene flow from other populations (Fig. 5). In particular, immigration to TM and IS was suggested to be very limited (Fig. 5b). No significant asymmetric pattern of gene flow was detected. In both sexes, individual heterozygosity did not show positive correlation with body size (Fig. 6).

Correlation test with Spearman’s rank correlation coefficient showed no significant correlation between cumulative human population density and observed heterozygosity (Fig. S1).

## Discussion

### Body size and sex ratio

In Japan, large males of *B. latro* are selectively harvested, which has caused the sex ratio to become skewed toward females and average male body size to become miniaturized in heavily harvested populations^38^. In the present study, sex ratio was significantly skewed toward females in populations IS and HT, suggesting that male-selective harvesting pressure is higher in IS and HT than in the other populations. However, past human population density was not correlated with body size, sex ratio and genetic diversities of *B. latro* (Fig. S1), suggesting that human population density does not simply reflect the intensity of fishery pressure on *B. latro*. Although adult male and female *B. latro* are expected to reach a thoracic length of 80 mm and 60 mm, respectively^29^, in the present study large males (>40 mm) were very rarely seen except for TM, even in the populations where the sex ratio was less, or not, skewed toward females. A lack of large males promotes resource reduction through negative impacts on life history parameters (e.g., decrease of mating opportunity)^39^. Our findings suggest that urgent implementation of an effective management policy is needed in Ryukyu Islands.

### Genetic diversity and gene flow

*Birgus latro* can live for 60 years^47^ or longer^48^. At 10 years of age, males and females generally have a thoracic length of around 30 mm and 25 mm, respectively^49^. Overharvesting of *B. latro* likely started in Japan after the end of the Second World War^37^, and although the need for conservation efforts was noted as early as the 1970s, we were told by residents of Okinawa that overharvesting has continued and even become more severe in the past two decades (Fujita, personal communication). Thus, most small individuals analysed in the present study (male, thoracic length <30 mm; female, <25 mm) would have been recruited after the intensification of overharvesting, whereas some of the largest individuals could have been recruited before. This suggests that since overharvesting reduces genetic diversity within a population^1–3^, the genetic diversity of small *B. latro* individuals should be lower than that of large individuals. However, in the present study, we did not find a correlation between thoracic length and population- or individual-level genetic diversity, as estimated by using COI gene and MIG-seq data. The average thoracic length of large individuals used in the COI gene analysis was 40 mm and 25 mm for males and females, respectively; therefore, based on the growth curve of *B. latro*, most of these individuals were recruited in the last 15–20 years^49^. The thoracic length of most individuals used for the MIG-seq analysis was smaller than 40 mm for both sexes; therefore, based on the growth curve of *B. latro*, most of these individuals were recruited within the last 20 years for males and within the last 30 years for females. It is therefore, suggests that not a few individuals included in the genetic analyses were likely recruited after the intensification of overharvesting and may have already suffered the impacts of overharvesting on genetic diversity. Growth rate can, however, vary among individuals based on resource availability and levels of social competition (e.g., growth can be slowed considerably when individuals lose a limb in combat). Further monitoring and analysis using more samples from each population with wide range of body size are needed to evaluate the effect of overharvesting on genetic diversity of *B. latro*.

To conserve genetic diversity, genetic mutation or immigration from other populations with high genetic diversity are needed^3^. Once genetic diversity is lost, recovery by genetic mutation takes many generations, and as both the coastal and oceanic environments are being rapidly changed by human activity^50–52^, mutation-based recovery is not realistic. In contrast, immigration-based recovery can occur relatively quickly^3^. In the COI analysis, no significant genetic differentiation between the study populations was found, and in the MIG-seq analysis, most population pairs showed no significant genetic differentiation. The pelagic zoeal larval stage of *B. latro* lasts for 18–23 days^53,54^, and megalopae settle at coastal areas and find a gastropod shell for migrating to land by around 10 days post settlement^55,56^. Therefore, the whole larval period lasts 4–5 weeks, which is similar to that of the coral *Acropora digitifera*^57^. It has been reported that population connectivity is generally high among *A. digitifera* populations in the Ryukyu Islands^58^, and the sampling locations in that study were similar to those in the present study. This suggests that the larval period of *B. latro* is long enough to allow sufficient larval dispersal among the study populations. This assumption is supported by the results of the Mantel test, which showed a non-significant correlation between genetic distance and geographic distance, which suggests that migration via larval dispersal frequently occurs among Japanese populations of *B. latro*.

Although a high gene flow has been maintained in *B. latro* populations in the Ryukyu Islands, MIG-seq analysis detected significant genetic differentiation between four population pairs: TM–MK, TM–YG, IS–MK, and IS–YG. This is consistent with the divMigrate results showing that immigration to TM and IS from other populations is limited and IE, MK, HT, YG, and IR have a role as core populations within the overall population in the Ryukyu Islands. Local water current can work as a dispersal barrier in Ryukyu islands^59^. In addition, TM is far from the large islands (Fig. 1). These factors may contribute to the limitation of immigration to TM and IS. Similarly, genetic differentiation has been shown between populations in the Indian and Pacific Oceans^60,61^ and between populations in the Ryukyu Islands and populations in Micronesia, Palau, and Indonesia^62^, indicating that immigration from the Indian Ocean, Micronesia, Palau, and Indonesia to the Ryukyu Islands is likely limited. All populations analyzed in the present study had high fixation index values, suggesting high rates of inbreeding and that a further reduction of effective population size driven by overharvesting and habitat degradation may reduce gene flows and drastically increase the risk of inbreeding depression.

Here, we report COI- and MIG-seq-based genetic diversity, sex ratio and body size distributions in 8 Japanese populations of *B. latro*. We found that 5 populations (IE, MK, HT, YG, and IR) that are important for the maintenance of the genetic diversity of the other populations via immigration through larval dispersal. Low genetic diversity can affect the fitness^6^ and ability of individuals to survive and adapt in future environments^3^. To conserve the genetic diversity of *B. latro* populations in Japan, and therefore to conserve *B. latro* as an important fishery resource, we recommend long-term monitoring of genetic diversity, sex ratio, and body size composition. In Okinawa prefecture (the administrative division that includes the Ryukyu Islands), several municipal governments have implemented regulations that prohibit the catch of *B. latro* of certain sizes; we hope that such regulation will be implemented soon across the whole region.

## Methods

### Field surveys and sample collection

Field surveys were conducted in Ie (IE), Miyako (MY), Kurima (MK), Minna (TM), Ishigaki (IS), Hatoma (HT), Iriomote (IR), and Yonaguni (YG) in the Ryukyu Islands, Japan (Fig. 1; Table 1). Individuals of *B. latro* were randomly collected by hand and the sex and thoracic length (as an index of body size) of each individual were recorded. For DNA analysis, part of the third pereiopod was also collected. Tissue samples were fixed and preserved in 99.5% ethanol. All crabs were released at the sampling site after tissue sampling. Field surveys and sample collection were conducted in accordance with the regulations of local governments in Okinawa Prefecture, Japan.

### Statistical analyses

We examined whether the sex ratio of each population is skewed toward either sex by using the χ^2^-test. In addition, we compared thoracic length among populations for each sex by using Tukey’s test. All analyses were performed using R v3.3.0^63^.

We assumed that the past human population density reflects the intensity of fishery pressure on *B. latro*. We therefore tested correlation between cumulative human population densities and, sex ratio, median body size, and genetic diversity indexes of *B. latro* in each island population with Spearman’s rank correlation coefficient. Data of every 5 year human population densities (1955–2015) of each island was obtained from Okinawa Prefecture^64^. We cumulated the human population density of 1955–2015 (Table 1), and used for the analyses.

### mtDNA COI gene sequencing

DNA was extracted from tissue samples by using a DNeasy Blood and Tissue Kit (Qiagen, Hilden, Germany). Partial sequences of the mtDNA COI-encoding region were amplified by polymerase chain reaction (PCR) with the universal primers LCO1490 and HCO2198^65^, as well as TaKaRa Ex Taq (TaKaRa, Shiga, Japan). The PCR conditions were as follows: initial denaturation at 94 °C for 2 min; 35 cycles each at 94 °C for 30 s, annealing at 47 °C for 30 s, and extension at 72 °C for 1 min; and a final extension step at 72 °C for 2 min. The PCR products were purified using Exo-SAP IT (Affymetrix, USB, Cleveland, USA) and sequenced using an Applied Biosystems 3730xl DNA Analyser and the same primers as used for the PCR.

### MIG-seq

Genome-wide single nucleotide polymorphisms (SNPs) were obtained using the protocol^45^. In brief, MIG-seq was used to amplify a few hundred to a few thousand genome-wide SNPs around ISSRs by using eight universal pairs of multiplex ISSR primers (MIG-seq primer set 1) for the first PCR. Then, DNA libraries with different indexes were pooled and sequenced by using a MiSeq system (sequencing control software v2.0.12, Illumina) and a MiSeq Reagent Kit v3 (150 cycle) (Illumina). A total of 83 individuals were analyzed in the MIG-seq analysis.

To eliminate low-quality reads and primer sequence reads from the raw data, we used the FASTX-Toolkit v0.0.14 (fastq_quality_filter) (http://hannonlab.cshl.edu/fastx_toolkit/index.html) with a fastq-quality-filter setting of –Q 33 –q 30 –p 40. We removed adapter sequences for the MiSeq run from both the 5′ end (GTCAGATCGGAAGAGCACACGTCTGAACTCCAGTCAC) and 3′ end (CAGAGATCGGAAGAGCGTCGTGTAGGGAAAGAC) by using Cutadapt v1.13^66^, and then excluded short reads less than 80 bp. The quality-filtered sequence data were demultiplexed and filtered through the software Stacks v1.46^67,68^. We used Stacks v1.4^68^ to stack the reads and extract SNPs. First, we used the U-stacks program with the following settings: ‘minimum depth of coverage required to create a stack (m)’ = 3, ‘maximum distance allowed between stacks (M)’ = 1, ‘maximum distance allowed to align secondary reads to primary stacks (N)’ = 1, with the deleveraging and removal algorithms enabled. Then, we used the C-stacks program with the option ‘number of mismatches allowed between sample loci when building the catalog (n)’ = 4, followed by the S-stacks program. Finally, we used the Populations program in Stacks v1.4 by restricting the data analysis to the criteria (the minimum percentage of individuals required to process a locus across all data was set at 50% and restricting the data analysis to a single SNP per locus. No locus was identified as an outlier using BayScan^69^.

## Population genetic analyses

### mtDNA COI gene analysis

Haplotype networks were constructed using the haploNet function in the R package ‘pegas’ v0.11^70^. Haplotype diversity and nucleotide diversity were calculated for each population by using Arlequin 3.5^71^. In addition, we pooled all sequence data for each sex and divided them into two size groups, small and large, with the small group including males with thoracic length < 30 mm and females with thoracic length < 25 mm (i.e., individuals roughly less than 10 years old)^49^, and calculated genetic diversities for the two size classes. Mean ± standard deviation of the thoracic length of each group is 23.6 ± 4.0 mm (N = 25), 35.1 ± 5.6 mm (N = 29), 23.2 ± 2.5 mm (N = 22), and 31.6 ± 3.9 mm (N = 62) for small male, large male, small female, and large female, respectively. Population pairwise PhiPT values were estimated using the Analysis of Molecular Variance (AMOVA) method and GenAlEx 6.5^72^ and tested for significance based on 999 permutations. Statistical significance levels for all pairwise tests were 0.05 after adjusting for multiple comparisons using false discovery rate (FDR) correction^73^.

### MIG-seq

Based on 495 SNP markers, ratio of the number of observed alleles, observed heterozygosity, expected heterozygosity, and fixation index were estimated using GenAlEx 6.5^72^. Pairwise population *F*_ST_ values were estimated by using the AMOVA method and GenAlEx 6.5. Statistical significance levels for all pairwise tests were 0.05 after adjusting for multiple comparisons using FDR correction^68^. Isolation by distance was tested using Mantel’s test based on 9999 permutations from the comparison of all pairwise *F*_ST_/(1 − *F*_ST_) values with pairwise geographic distances in kilometers (straight-line distance) using GenAlEx 6.5^72^. In addition, individual heterozygosity was estimated using GENHET v2.3^74^ with R v3.3.0^63^.

The gene flow among populations was estimated by using divMigrate-online^75^ (https://popgen.shinyapps.io/divMigrate-online/). This program produces a migration network graph with relative values for gene flow among populations scaled to the largest magnitude estimated. For the analysis, we selected 61 MIG-seq markers that were detected in at least 68% of the samples. We used *N*_M_ as a measure of genetic distance. The significance of asymmetrical gene flow among populations was tested using 1000 bootstrap iterations.

## Supplementary information

Figure S1.

Table S1.

Table S2.

Table S3.

Dataset1.

## Data Availability

Raw data of body length and sex ratio of *B. latro* are available in supplementary information. mtDNA COI sequences (Accession nos. LC479132- LC479285) and law data of MIG-seq (Accession no. PRJDB8390) were deposited in DNA Data Bank of Japan (DDBJ).
