# Supplementary material for: Evaluating the effect of overharvesting on genetic diversity and genetic population structure of the coconut crab
Source: Sci Rep. 2020 Jun 22;10:10026. doi: 10.1038/s41598-020-66712-4 (PMC7308380; doi:10.1038/s41598-020-66712-4)
Supplement: Supplementary file 1 — Figure S1. [file 41598_2020_66712_MOESM1_ESM.pdf]

Supplementary information

Evaluating the effect of overharvesting on genetic diversity and population genetic structure of the coconut crab

Takefumi Yorisue[1, 2 † *], Akira Iguchi[2 †], Nina Yasuda[3], Yuki Yoshioka[4], Taku Sato[5], Yoshihisa Fujita[6]

[†] These authors contributed equally to this work
*correspondence
Email: yorisue@gmail.com

1. Integrative Aquatic Biology, Onagawa Field Center, Graduate School of Agricultural Science, Tohoku University, 3-1 Mukai, Konori-hama, Onagawa, Oshika, Miyagi 986-2242, Japan
2. Marine Geo-Environment Research Group, Institute of Geology and Geoinformation, National Institute of Advanced Industrial Science and Technology (AIST), AIST Tsukuba Central 7, 1-1-1 Higashi, Tsukuba, Ibaraki 305-8567, Japan
3. Department of Marine Biology and Environmental Science, Faculty of Agriculture, University of Miyazaki, Gakuenkibana-dai Nishi 1-1, Miyazaki 889-2192, Japan
4. Department of Bioresources Engineering, National Institute of Technology, Okinawa College, 905, Henoko, Nago, Okinawa 905-2192, Japan
5. Research Center for Marine Invertebrates, National Research Institute of Fisheries and Environment of Inland Sea, Japan Fisheries Research and Education Agency, Momoshima, Onomichi, Hiroshima 722-0061, Japan
6. Okinawa Prefectural University of Arts, 1-4, Shuri Tonokura-cho, Naha-shi, Okinawa 903-8602, Japan

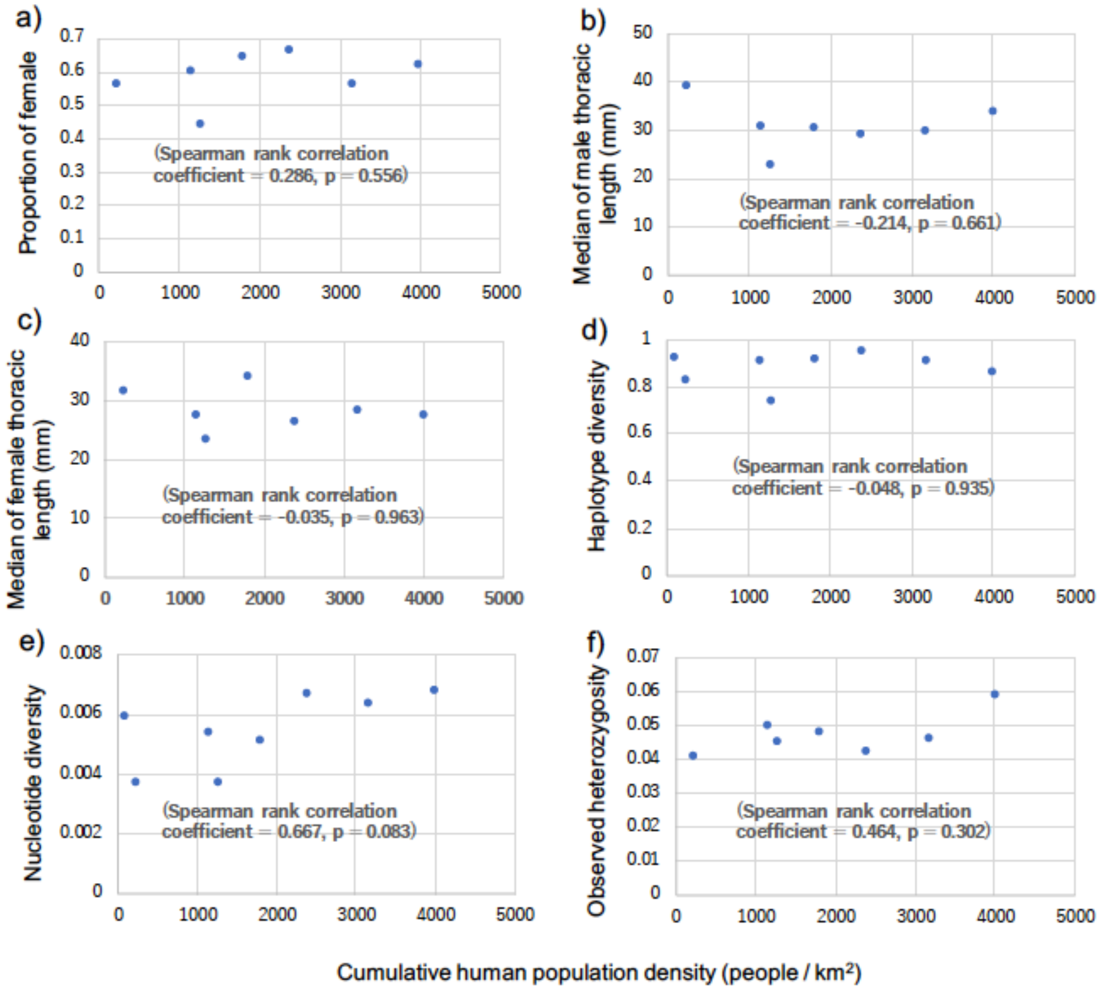

Fig.S1. Relationships between cumulative human population density of each islands and (a)proportion of male, (b)median of male thoracic length, (c) median of female thoracic length, (d)COI-based haplotype diversity,(e)COI-based nucleotide diversity, (f)MIG-seq-based observed heterozygosity of *B. latro* populations.
