# Supplementary material for: Evaluating the effect of overharvesting on genetic diversity and genetic population structure of the coconut crab
Source: Sci Rep. 2020 Jun 22;10:10026. doi: 10.1038/s41598-020-66712-4 (PMC7308380; doi:10.1038/s41598-020-66712-4)
Supplement: Supplementary file 2 — Table S1. [file 41598_2020_66712_MOESM2_ESM.docx]

**Supplementary information**

**Evaluating the effect of overharvesting on genetic diversity and population genetic structure of the coconut crab**

Takefumi Yorisue^1, 2†*^, Akira Iguchi^2†^, Nina Yasuda^3^, Yuki Yoshioka^4^, Taku Sato^5^, Yoshihisa Fujita^6^

^†^These authors contributed equally to this work

*correspondence

Email: yorisue@gmail.com

1. Integrative Aquatic Biology, Onagawa Field Center, Graduate School of Agricultural Science, Tohoku University, 3-1 Mukai, Konori-hama, Onagawa, Oshika, Miyagi 986-2242, Japan
2. Marine Geo-Environment Research Group, Institute of Geology and Geoinformation, National Institute of Advanced Industrial Science and Technology (AIST), AIST Tsukuba Central 7, 1-1-1 Higashi, Tsukuba, Ibaraki 305-8567, Japan
3. Department of Marine Biology and Environmental Science, Faculty of Agriculture, University of Miyazaki, Gakuenkibana-dai Nishi 1**-**1, Miyazaki 889**-**2192, Japan
4. Department of Bioresources Engineering, National Institute of Technology, Okinawa College, 905, Henoko, Nago, Okinawa 905-2192, Japan
5. Research Center for Marine Invertebrates, National Research Institute of Fisheries and Environment of Inland Sea, Japan Fisheries Research and Education Agency, Momoshima, Onomichi, Hiroshima 722-0061, Japan
6. Okinawa Prefectural University of Arts, 1-4, Shuri Tonokura-cho, Naha-shi, Okinawa 903-8602, Japan

Table S1. *P* values of Tukey’s test for body size composition in female (above) and male (below) *B. latro* populations. Bold indicates *P* < 0.05.

|  | IE | M | MK | TM | I | H | YG |
| --- | --- | --- | --- | --- | --- | --- | --- |
| IE |  | 0.987 | **0.000** | 0.599 | 0.653 | 0.047 | 0.946 |
| M | 0.676 |  | 0.270 | 0.397 | 1.000 | 0.051 | 1.000 |
| MK | 0.165 | **0.020** |  | **0.000** | **0.026** | **0.000** | 0.076 |
| TM | **0.001** | 0.998 | **0.000** |  | **0.000** | 0.336 | 0.079 |
| I | 0.996 | 0.386 | 0.579 | **0.000** |  | **0.000** | 1.000 |
| H | 1.000 | 0.778 | **0.040** | **0.002** | 0.964 |  | **0.003** |
| YG | 1.000 | 0.724 | **0.339** | **0.014** | 0.998 | 1.000 |  |
