# Supplementary material for: Evaluating the effect of overharvesting on genetic diversity and genetic population structure of the coconut crab
Source: Sci Rep. 2020 Jun 22;10:10026. doi: 10.1038/s41598-020-66712-4 (PMC7308380; doi:10.1038/s41598-020-66712-4)
Supplement: Supplementary file 3 — Table S2. [file 41598_2020_66712_MOESM3_ESM.docx]

**Supplementary information**

**Evaluating the effect of overharvesting on genetic diversity and population genetic structure of the coconut crab**

Takefumi Yorisue^1, 2†*^, Akira Iguchi^2†^, Nina Yasuda^3^, Yuki Yoshioka^4^, Taku Sato^5^, Yoshihisa Fujita^6^

^†^These authors contributed equally to this work

*correspondence

Email: yorisue@gmail.com

1. Integrative Aquatic Biology, Onagawa Field Center, Graduate School of Agricultural Science, Tohoku University, 3-1 Mukai, Konori-hama, Onagawa, Oshika, Miyagi 986-2242, Japan
2. Marine Geo-Environment Research Group, Institute of Geology and Geoinformation, National Institute of Advanced Industrial Science and Technology (AIST), AIST Tsukuba Central 7, 1-1-1 Higashi, Tsukuba, Ibaraki 305-8567, Japan
3. Department of Marine Biology and Environmental Science, Faculty of Agriculture, University of Miyazaki, Gakuenkibana-dai Nishi 1**-**1, Miyazaki 889**-**2192, Japan
4. Department of Bioresources Engineering, National Institute of Technology, Okinawa College, 905, Henoko, Nago, Okinawa 905-2192, Japan
5. Research Center for Marine Invertebrates, National Research Institute of Fisheries and Environment of Inland Sea, Japan Fisheries Research and Education Agency, Momoshima, Onomichi, Hiroshima 722-0061, Japan
6. Okinawa Prefectural University of Arts, 1-4, Shuri Tonokura-cho, Naha-shi, Okinawa 903-8602, Japan

Table S2. *Birgus latro* pairwise population values of PhiPT (below) and *F*_ST_ (above) via AMOVA. PhiPT and *F*_ST_ values were estimated based on COI and MIG-seq markers, respectively. Significance levels for all pairwise tests were *P* < 0.05 after adjusting for multiple comparisons using FDR correction. Significant values (*P* < 0.05) are shown in bold.

|  | IE | M | MK | TM | I | H | IR | YG |
| --- | --- | --- | --- | --- | --- | --- | --- | --- |
| IE |  | 0.011 | 0.008 | 0.022 | 0.035 | 0.002 | 0.008 | 0.023 |
| M | 0.005 |  | 0.000 | 0.049 | 0.076 | 0.000 | 0.000 | 0.002 |
| MK | 0.010 | 0.000 |  | **0.074** | **0.076** | 0.000 | 0.008 | 0.000 |
| TM | 0.000 | 0.000 | 0.000 |  | 0.000 | 0.033 | 0.044 | **0.093** |
| I | 0.000 | 0.000 | 0.000 | 0.000 |  | 0.023 | 0.050 | **0.104** |
| H | 0.018 | 0.000 | 0.000 | 0.000 | 0.000 |  | 0.000 | 0.000 |
| IR | 0.003 | 0.000 | 0.000 | 0.000 | 0.000 | 0.000 |  | 0.007 |
| YG | 0.005 | 0.000 | 0.000 | 0.000 | 0.000 | 0.000 | 0.000 |  |
