# Supplementary material for: Evaluating the effect of overharvesting on genetic diversity and genetic population structure of the coconut crab
Source: Sci Rep. 2020 Jun 22;10:10026. doi: 10.1038/s41598-020-66712-4 (PMC7308380; doi:10.1038/s41598-020-66712-4)
Supplement: Supplementary file 4 — Table S3. [file 41598_2020_66712_MOESM4_ESM.docx]

**Supplementary information**

**Evaluating the effect of overharvesting on genetic diversity and population genetic structure of the coconut crab**

Takefumi Yorisue^1, 2†*^, Akira Iguchi^2†^, Nina Yasuda^3^, Yuki Yoshioka^4^, Taku Sato^5^, Yoshihisa Fujita^6^

^†^These authors contributed equally to this work

*correspondence

Email: yorisue@gmail.com

1. Integrative Aquatic Biology, Onagawa Field Center, Graduate School of Agricultural Science, Tohoku University, 3-1 Mukai, Konori-hama, Onagawa, Oshika, Miyagi 986-2242, Japan
2. Marine Geo-Environment Research Group, Institute of Geology and Geoinformation, National Institute of Advanced Industrial Science and Technology (AIST), AIST Tsukuba Central 7, 1-1-1 Higashi, Tsukuba, Ibaraki 305-8567, Japan
3. Department of Marine Biology and Environmental Science, Faculty of Agriculture, University of Miyazaki, Gakuenkibana-dai Nishi 1**-**1, Miyazaki 889**-**2192, Japan
4. Department of Bioresources Engineering, National Institute of Technology, Okinawa College, 905, Henoko, Nago, Okinawa 905-2192, Japan
5. Research Center for Marine Invertebrates, National Research Institute of Fisheries and Environment of Inland Sea, Japan Fisheries Research and Education Agency, Momoshima, Onomichi, Hiroshima 722-0061, Japan
6. Okinawa Prefectural University of Arts, 1-4, Shuri Tonokura-cho, Naha-shi, Okinawa 903-8602, Japan

Table S3. Summary of genetic diversity of *B. latro* based on mtDNA COI and MIG-seq analyses. *h*: haplotype diversity, π: nucleotide diversity, Na: ratio of the number of observed alleles, *H*_O_: observed heterozygosity, *H*_E_: expected heterozygosity, *F*: fixation index. Values in parenthese indicate standard deviation and standard error for mtDNA COI- and MIG-seq- based indices, respectively.

| Population | mtDNA COI | | MIG-seq | | | | |
| --- | --- | --- | --- | --- | --- | --- | --- |
|  | *h* | π |  | Na | *H*_O_ | *H*_E_ | *F* |
| IE | 0.9091 (0.0336) | 0.00629 (0.00381) |  | 1.390 (0.022) | 0.046 (0.005) | 0.099 (0.006) | 0.500 (0.024) |
| M | 0.8590 (0.0886) | 0.00677 (0.00421) |  | 1.263 (0.020) | 0.059 (0.007) | 0.090 (0.007) | 0.294 (0.028) |
| MK | 0.7391 (0.0587) | 0.00366 (0.00247) |  | 1.434 (0.022) | 0.045 (0.005) | 0.098 (0.006) | 0.460 (0.022) |
| TM | 0.8286 (0.0643) | 0.00364 (0.00253) |  | 1.228 (0.020) | 0.041 (0.006) | 0.080 (0.007) | 0.425 (0.027) |
| I | 0.9485 (0.0435) | 0.00663 (0.00405) |  | 1.265 (0.020) | 0.042 (0.005) | 0.086 (0.007) | 0.420 (0.025) |
| H | 0.9130 (0.0388) | 0.00507 (0.00319) |  | 1.499 (0.022) | 0.048 (0.005) | 0.115 (0.006) | 0.532 (0.022) |
| IR | 0.9231 (0.0604) | 0.00587 (0.00372) |  | na | na | na | na |
| YG | 0.9094 (0.0331) | 0.00533 (0.00333) |  | 1.501 (0.022) | 0.050 (0.005) | 0.108 (0.006) | 0.469 (0.022) |
| Large Female | 0.8557 (0.0324) | 0.00469 (0.00292) |  | na | na | na | na |
| Small Female | 0.9338 (0.0393) | 0.00587 (0.00366) |  | na | na | na | na |
| Large Male | 0.9097 (0.0297) | 0.00557 (0.00341) |  | na | na | na | na |
| Small Male | 0.8767 (0.0437) | 0.00585 (0.00358) |  | na | na | na | na |
